# A Comparative Study on the Properties of Rosin-Based Epoxy Resins with Different Flexible Chains

**DOI:** 10.3390/polym15214246

**Published:** 2023-10-28

**Authors:** Lianli Deng, Zehua Wang, Bailu Qu, Ying Liu, Wei Qiu, Shaohe Qi

**Affiliations:** 1 Hunan Provincial Key Laboratory of Xiangnan Rare-Precious Metals Compounds and Applications, School of Chemistry and Environmental Science, Xiangnan University, Chenzhou 423000, China; lianlideng@xnu.edu.cn (L.D.);; 2 School of Chemistry & Chemical Engineering, Guangxi University, Nanning 530004, China; 3 Changsha Ecological and Environmental Monitoring Centre of Hunan Province, Changsha 410001, China; 4 School of Chemical Engineering, Guizhou Institute of Technology, Guiyang 550003, China

**Keywords:** rosin, flexible chain, film property, bio-based epoxy, curing agent

## Abstract

This study aims to reveal the effects of flexible chain lengths on rosin-based epoxy resin’s properties. Two rosin-based epoxy monomers with varying chain lengths were synthesized: AR-EGDE (derived from ethylene glycol diglycidyl ether-modified acrylic acid rosin) and ARE (derived from acrylic acid rosin and epichlorohydrin). Diethylenetriamine (DETA), triethylenetetramine (TETA), and tetraethylenepentamine (TEPA) with different flexible chain lengths were used as curing agents. The adhesion, impact, pencil hardness, flexibility, water and heat resistance, and weatherability of the epoxy resins were systematically examined. It was found that when the flexible chains of rosin-based epoxy monomers were grown from ARE to AR-EGDE, due to the increased space of rosin-based fused rings, the toughness, adhesion, and water resistance of the rosin-based epoxy resins were enhanced, while the pencil hardness and heat resistance decreased. However, when the flexible chains of curing agents were lengthened, the resin’s performance did not change significantly because the space between the fused rings changed little. This indicates that the properties of the rosin-based resins can only be altered when the introduced flexible chain increases the space between the fused rings. The study also compared rosin-based resins to E20, a commercial petroleum-based epoxy of the bisphenol A type. The rosin-based resins demonstrated superior adhesion, water resistance, and weatherability compared to the E20 resins, indicating the remarkable durability of the rosin-based resin.

## 1. Introduction

Epoxy resins are three-dimensional network structure polymers formed by the reaction of epoxy monomer and a curing agent; thus, the properties of epoxy resins depend on the structure of the epoxy monomer and curing agent [1]. Consequently, epoxy resins containing polar groups such as hydroxyl and ether feature excellent mechanical strength, good dielectric properties, chemical resistance, strong adhesion, low shrinkage, and ease of processability [2,3], allowing for their widespread applications in different fields such as adhesives, coatings, composites, construction, and electronics. However, almost 70% of the world’s production of epoxy resin is derived from bisphenol A (BPA), a petroleum-based compound [4,5]. Using renewable resources instead of BPA can protect petrochemicals, fix CO_2_, accelerate the C-cycle process, and reduce greenhouse gases, all the while meeting society’s requirements. A number of studies have demonstrated that humans could overcome the resource problem by utilizing just seven percent of the biomass that exists [6].

Rosin, a renewable resource that is abundantly available, is produced from pine and conifer trees, with a yearly global yield of approximately 1.27 million tons [7,8]. It is a mixture containing around 75% rosin acid, and the rosin acid molecules consist of a fused ring and active functional groups, such as double bonds and a carboxyl group, which are easily chemically modified. Diels–Alder type reactions produce rosin derivatives such as fumaropimaric acid (FPA), acrylicpimaricacid (APA), maleopimaric acid (MPA), and methyl maleopidate (MMP). Owing to their structural characteristics, low cost, biodegradability, biocompatibility, and corrosion resistance, rosin and its derivatives are valuable feedstocks for an array of polymers [9,10] and have found a broad range of applications [8], such as coatings [11,12,13,14,15,16,17,18], packaging [19,20], electrical equipment [6], antibacterial and antiviral polymers [19,21,22,23,24], surfactants, and adhesives [25,26,27,28,29]. Hence, the replacement of BPA with rosin and its derivatives as a fossil feedstock has gained increasing attention [4,5,30,31,32,33]. It has been demonstrated that the mechanical and thermal properties of rosin-based epoxy resin are comparable to those of bisphenol A epoxy resin, due to the analogous rigidity of the fused ring of rosin and the benzene ring of bisphenol A. It enables rosin to be a potential partial or full substitute for petroleum-based epoxy monomers and curing agents [2,30,34,35,36,37,38,39,40,41,42]. On the other hand, in order to improve the mechanical and thermal properties of bio-based epoxy resins and vitrimers such as vegetable oil-based epoxy resins that meet the application requirements of industry, rosin-based derivatives are often introduced as epoxy monomers or curing agents [43,44]. However, the fused ring of rosin-based epoxy resin is not only rigid but also brittle, leading to brittle fractures in reaction to external pressures and a decrease in its mechanical properties. For example, MPA (rosin-based curing agents) was employed by Liu et al. as a co-curing agent for DGEBA. As the MPA content grew, the material’s mechanical parameters fell. The flexible chain was incorporated into the rosin-based epoxy resin to reduce the brittleness of the rosin-based fused ring to obtain a resin with improved performance. The effect of the quantity of flexible chains on the mechanical properties of rosin-based epoxy resin was studied in our previous research [45]. It was shown that the introduction of appropriate flexible chains into the rosin-based epoxy monomer can reduce brittleness and increase toughness, thereby improving its mechanical properties. Huang et al. combined the rigid rosin epoxy monomer with a flexible dimer acid epoxy monomer, producing resins with enhanced mechanical and thermal properties [46]. Li et al. combined curing agents derived from rigid rosin and flexible fatty acids to achieve balanced mechanical and thermal properties [47]. 

However, to our knowledge, the effects of epoxy monomers and curing agents with different flexible chain lengths on the properties of rosin-based epoxy resins have not been studied systematically, nor have the effects of flexible chains with different structures been compared. In this study, rosin-based epoxy monomers with various flexible chains were obtained: ARE, a diglycidyl ester derived from acrylic acid rosin and epichlorohydrin that lacked flexible chains, and AR-EGDE, an ethylene glycol diglycidyl ether-modified acrylic acid rosin with flexible chains. Diethylenetriamine (DETA), triethylenetetramine (TETA), and tetraethylenepentamine (TEPA) with different chain lengths were used concurrently to cure the aforementioned epoxy resins. The impacts of epoxy monomers and curing agents with various flexible chains on the adhesion, impact, pencil hardness, flexibility, water and heat resistance, and weatherability of rosin-based epoxy resins were studied. In the meantime, the effects of two distinct flexible chains on the properties of rosin-based epoxy resin were compared, and the structure of the flexible chain required to increase its toughness was determined. In addition, commercial epoxy resin was utilized for comparison purposes. According to the results, the bio-based resin with superior adhesion outperforms the commercial one (DGEBA).

## 2. Experimental

### 2.1. Materials

Wuzhou Sun Shine Forestry & Chemicals Co., Ltd. (Wuzhou, China) provided the acrylic acid rosin (ARA) (238 mg KOH·g^−1^). Ethylene glycol diglycidyl ether (EGDE) with an epoxy value of 0.73 mol/100 g was acquired from Wuhan Yuancheng Create Technology Co. (Wuhan, China) E-20 (DGEBA with an epoxy value of 0.20 mol/100 g) was purchased from Wuxi Resin Factory of Blue Star New Chemical Material Co., Ltd. (Wuxi, China). From Sinopharm Group Chemical Reagent Co., Ltd. (Shanghai, China), diethylenetriamine (DETA) and triethylenetetramine (TETA) were purchased. Epichlorohydrin (EC), ethanol (EtOH), acetone, potassium hydroxide (KOH), and triethylamine (Et_3_N) were of chemically pure quality and were utilized directly.

### 2.2. Preparation of Epoxy Monomers

#### 2.2.1. ARE, Diglycidyl Ester from ARA (Figure 1) 

ARA (100.00 g; 0.42 mol of carboxyl group), EC (156.67 g; 1.68 mol of epoxy group), and Et_3_N (0.51 g; 0.2 wt% of the total weight of ARA and EC) were added to a 500 mL four-neck round-bottom flask equipped with a reflux condenser, a mechanical stirrer, a thermometer, and an N_2_ inlet. The temperature of the system was raised to 110 °C and maintained until the acid number declined below 0.5 mg KOH/g. After cooling to 70 °C, 0.42 mol of solid NaOH (17.00 g) was added, and the temperature was maintained for 3 h. After the reaction, the precipitate was filtered out, and the filtrate was neutralized with water. After separation, the organic layer was evaporated at approximately 50 °C to recover excess epichlorohydrin, resulting in a yellow, transparent, viscous liquid.

**Figure 1:**
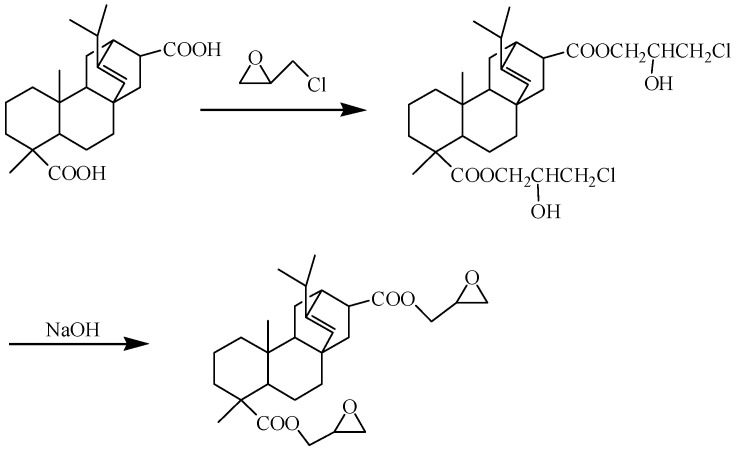
Synthesis route of ARE.

#### 2.2.2. AR-EGDE, the Copolymers of Ethylene Glycol Diglycidyl Ether-Modified ARA (Figure 2)

EGDE (116.50 g, 0.85 mol of epoxy group) and ARA (50 g, 0.21 mol of carboxyl group) were added to a 500 mL four-necked flask equipped with a reflux condenser, agitator, thermometer, and nitrogen tubing. Et_3_N (0.02% of the ARA mass) was added as a catalyst once the acrylic rosin had been completely dissolved. After reacting at 130 °C for 1 h, an additional 50.00 g of ARA (0.2117 mol carboxyl group) was added, and the reaction temperature was maintained until the acid value fell below 0.5 mg KOH/g, at which point the reaction was terminated. The product was a yellow, viscous, transparent liquid.

**Figure 2:**
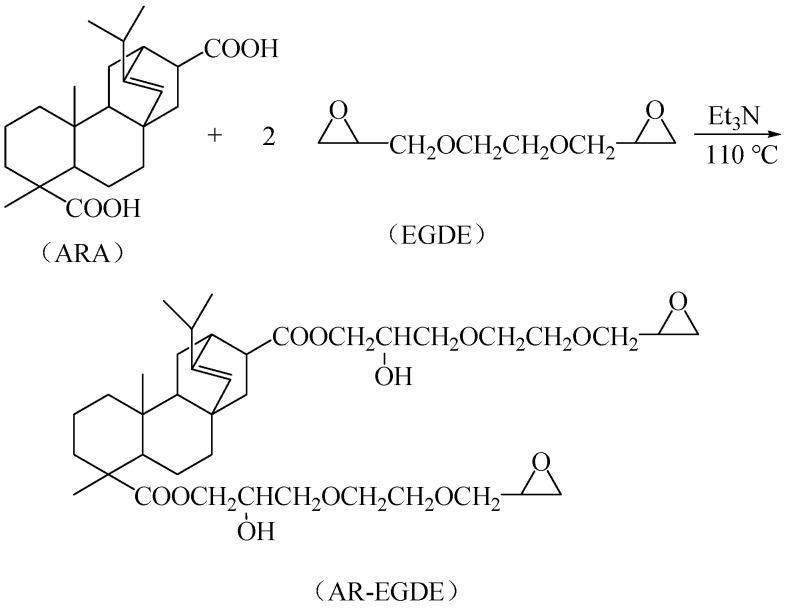
Synthesis route of AR-EGDE.

### 2.3. Preparation of Test Samples

**Figure 3:**
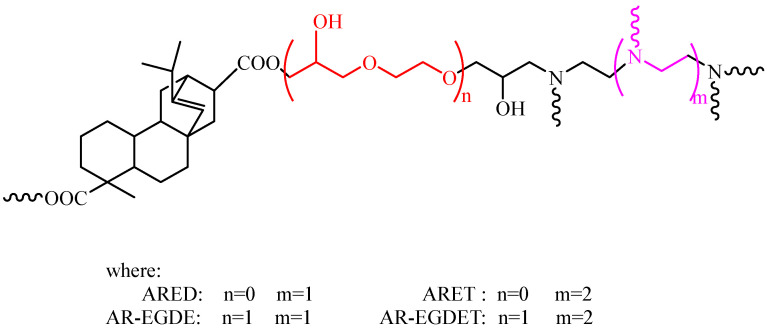
Rosin-based epoxy resins with different flexible chains.

Stoichiometrically, the epoxy monomers and curing agents were blended. Then, certain amounts of mixed solvents (xylene and butanol) were added to the above blends. The ratio of xylene to butanol by mass was 70:30. The method yielded a mixture that was aged for 30 min before being coated on 120 mm × 50 mm tinplates. This was carried out in accordance with GB/T 1727-2021 [48]. For each test, at least three tinplates were prepared. The formulations for the epoxy resins are listed in Table 1, and Figure 3 depicts the structure of epoxy resin based on rosin. Simultaneously, the epoxy monomer and curing agent were theoretically combined to produce 80 mm × 10 mm × 4 mm strip resin samples. The curing procedure required 12 h at room temperature, followed by 4 h at 100 °C in the oven. 

### 2.4. Characterization

Using the KBr method, an RFX-65A (Analect) FTIR instrument was used to acquire the infrared spectra of the prepared epoxy monomers. 

The hardness was assessed in accordance with GB/T 6739-2006 [49]. 

Using the paint film scriber A2012058 (Elcometer), the adhesion was evaluated using a cross-cut test per GB/T 9286-2021 [50]. 

The impact resistance was evaluated in accordance with GB/T 1732-2020 [51], using a 20 cm impact height and a 1 kg mallet. 

The flexibility was determined via GB/T 1731-2020 [52]. 

The heat resistance was measured as per GB/T 1735-2009 [53]. The tinplates were placed in an oven at 100 °C for 3 days. They were cooled to room temperature and examined for color changes or other signs of coating deterioration. 

The tinplates were placed in the sun for 60 days to test weatherability. 

For 49 days, painted tinplates were immersed vertically in 3000 mL water-filled glass beakers to study the water resistance behavior of the films. Regular inspections of the tinplates were conducted to assess the level of attack on the paint coatings and the substrate. At various intervals, the tinplates were removed, rinsed with water, and visually inspected for film integrity, overall appearance, and film failure.

The swelling behavior was investigated by immersing strip-shaped samples in water at room temperature. The percent change in mass was obtained using the following equation: percent change in mass = (W_2_ − W_1_)/W_1_ × 100%, where W_2_ and W_1_ are the weights after and before absorption, respectively.

## 3. Results

### 3.1. Characterization of Epoxy Resin Monomers 

**Figure 4:**
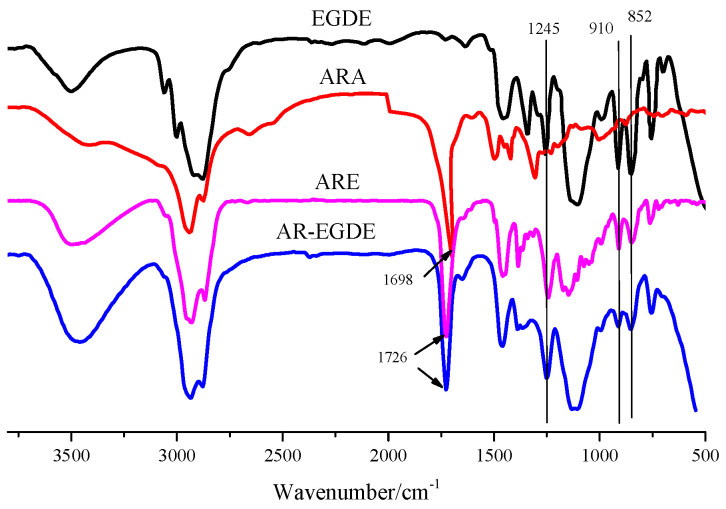
FTIR spectra of EGDE, ARA, ARE, and AR-EGDE.

By reacting the carboxyl groups of ARA with EC or EGDE, respectively, it is possible to generate ester-containing ARE and AR-EGDE. Figure 1 and Figure 2 depict the formulations of the chemical reactions. The FTIR spectra of the EGDE, ARA, and epoxy monomers (AR-EGDE and ARE) are shown in Figure 4. For ARE and AR-EGDE, the broad absorption peaks attributed to the -COOH of ARA between 3000 and 3500 cm^−1^ and around 1698 cm^−1^ nearly disappeared. Simultaneously, the conspicuous absorption peaks at 1726 cm^−1^, ascribed to the elongation of carbonyl groups in the ester groups are clearly visible. This indicates that the ARE and AR-EGDE formed. The epoxy groups were also confirmed by absorption bands at 1245, 910, and 852 cm^−1^. The acid number of the reaction mixture decreased over the course of the reaction, reaching 0.5 mg KOH/g at the conclusion. This also demonstrated the esterification of the carboxyl and epoxy groups. In addition, the epoxy values for ARE and AR-EGDE were 0.35 mol·100 g^−1^ and 0.20 mol·100 g^−1^, respectively.

### 3.2. Film Properties of the Rosin-Based Epoxy Resins with Different Flexible Chains

The rigidity and brittleness of a rosin-based fused ring structure will have distinct effects on the material properties [45]. Epoxy resin film properties, such as the pencil hardness, adhesion, flexibility, and impact resistance, are listed in Table 2.

#### 3.2.1. The Effects of Epoxy Monomer Flexible Chain on the Film Properties of Rosin-Based Epoxy Resin

As shown in Table 3, the hardness of the ARE coatings (ARED and ARET) was 3H. Upon replacing ARE with AR-EGDE, the hardness of AR-EGDED and AR-EGDET decreased to 2H when the epoxy monomer flexible chain length was increased. This is due to the presence of more rosin-based fused rings in the former. The rigidity of the rosin-based fused ring results in an increase in the hardness value as its content increases. However, the adhesion test results were the opposite of the hardness test results. The evaluation for adhesion, which refers to the capacity of the coating to adhere to the substrate, was conducted utilizing the cross-cut test. This test involves the classification of adhesion on a numerical scale ranging from 0 to 5. A smaller numerical value is indicative of superior adhesion. For the AR-EGDE coatings with flexible chains, the grade was 0, with no signs of damage, while for the ARE coatings, the grade was 2-3, with flaking of about 5–35 percent. The potential reason for this phenomenon can be attributed to the inherent characteristics of the rosin-based fused ring structure, which possesses materials of both rigidity and brittleness. The presence of brittleness has a negative impact on the level of adhesion. The incorporation of a flexible chain into rosin-based epoxy resin leads to a reduction in brittleness, thereby resulting in enhanced adhesion properties [45,54]. In order to find out the reason, the flexibility and impact resistance of the epoxy coatings were tested. The AR-EGDE coatings with flexible chains have better flexibility and impact resistance than the ARE coatings(see Table 3). It suggests that introducing a flexible chain of glycidyl ether into rosin epoxy monomers increases the toughness and impact resistance of rosin-based epoxy coatings, and thus improves adhesion.

#### 3.2.2. The Effects of Curing Agent Flexible Chains on the Film Properties of Rosin-Based Epoxy Resin

The test results of pencil hardness, adhesion, flexibility, and impact resistance of rosin-based epoxy resins remained nearly identical when the epoxy monomers remained unchanged, but the curing agent was switched from DETA, which possesses a short flexible chain, to TETA, which possesses a long flexible chain. For instance, the ARE coatings exhibited a pencil hardness value of 2H, a cross-cut test grade of 3, and failed in the flexibility and impact resistance tests, regardless of the type of curing agent used. The above results show that the use of the amine curing agents, which possess flexible chains of longer lengths, does not improve the brittleness of the rosin-based fused ring. Consequently, it does not lead to enhancements in impact resistance, flexibility, and adhesion while reducing the hardness of the rosin-based epoxy resin.

#### 3.2.3. A Comparison of Film Properties between the Rosin-Based Epoxy Resin and Bisphenol A Epoxy Resin

The film properties of bisphenol A epoxy (E20) resin were also found to be unaffected by the flexible chain length of its curing agent. The pencil hardness of the E20 coatings was 3H, which was equivalent to that of the ARE coatings. This indicates that the rosin-based fused ring structure is comparable to the rigidity of the benzene ring of the bisphenol A type. The results of the E20 coating tests for flexibility and impact resistance were superior to those of ARE, indicating that the brittleness of rosin-based fused rings is greater than that of benzene rings. However, the cross-test grades of the E20 coatings were 5, which were not only lower than the AR-EGDE coatings with flexible chains, but also lower than that of the ARE coating, which indicates that the rosin-based fused ring has better adhesion than the benzene ring of bisphenol A epoxy resin. 

### 3.3. Water Resistance Tests of the Rosin-Based Epoxy Resin with Different Flexible Chain Lengths

**Figure 5:**
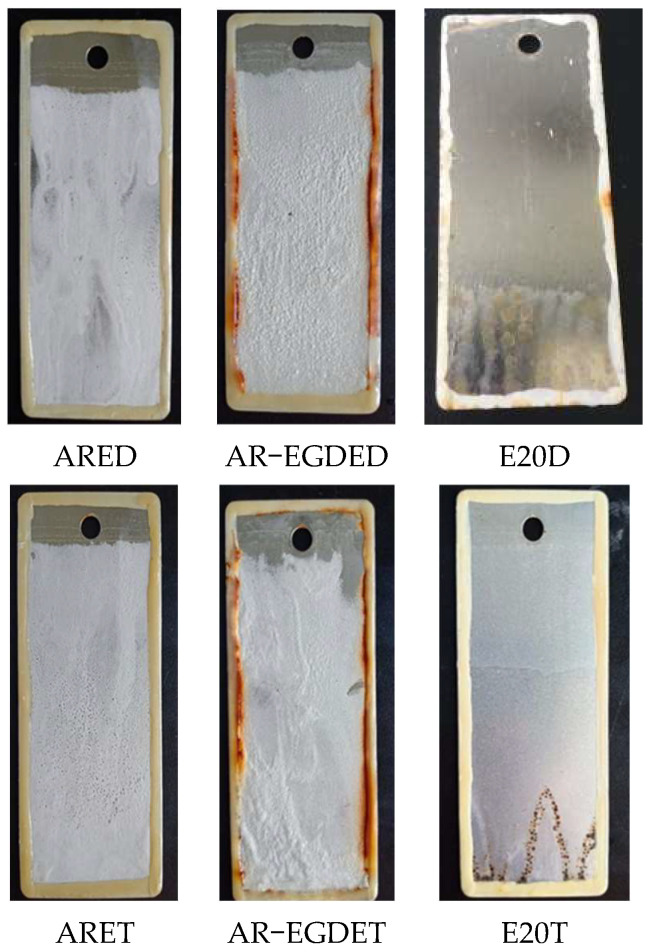
The images of immersion tests with water for 49 days.

**Figure 6:**
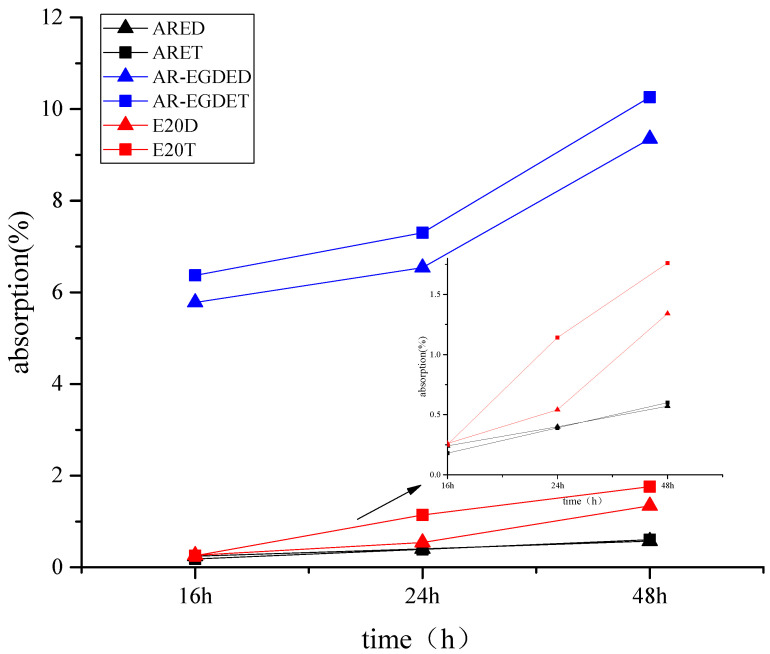
Water absorption rates of cured resins.

Figure 5 shows test images of tinplates immersed in water. The ARE epoxy coating without flexible chains turned white, whereas the AR-EGDE epoxy coating with flexible chains became wrinkly. This indicates that the water resistance of the rosin-based epoxy coating decreases as the flexible chain length of the epoxy monomers increases [55]. The reason for this is that as the flexible chain increases, the rigidity of the rosin-based fused ring decreases, and the cross-linking density of AR-EGDE resins decreases. Moreover, the flexible chain of the epoxy monomers contains hydroxyl groups and ether bonds that can form hydrogen bonds with water (see Figure 3). This conclusion was also supported by the water absorption tests of cured resins. In Table 3 and Figure 6, the AR-EGDE resins had 10 times higher water absorption rates than the ARE resins. When the epoxy monomers were identical and the curing agents were different, such as ARED and ARET, AR-EGDED and AR-EGDET, there was little variation in the water resistance of the resins. This demonstrates that the length of fatty amine curing agents has little effect on the water resistance of epoxy coatings.

There was no whitening phenomenon observed in E20 epoxy coatings, but there was slight rusting. The water resistance of the E20 epoxy coatings was even worse than that of the AR-EGDE from a rust perspective. In most cases, a material’s low water resistance results from its high water absorption. Nevertheless, the water absorption rates of the E20 coatings were significantly lower than those of AR-EGDE coatings (Figure 6). This suggests that the fused ring structure of the AR-EGDE resins is more resistant to water than the benzene ring of bisphenol A types, which should be related to their superior adhesion.

### 3.4. Heat Resistance of the Rosin-Based Epoxy Resin with Different Flexible Chain Lengths

**Figure 7:**
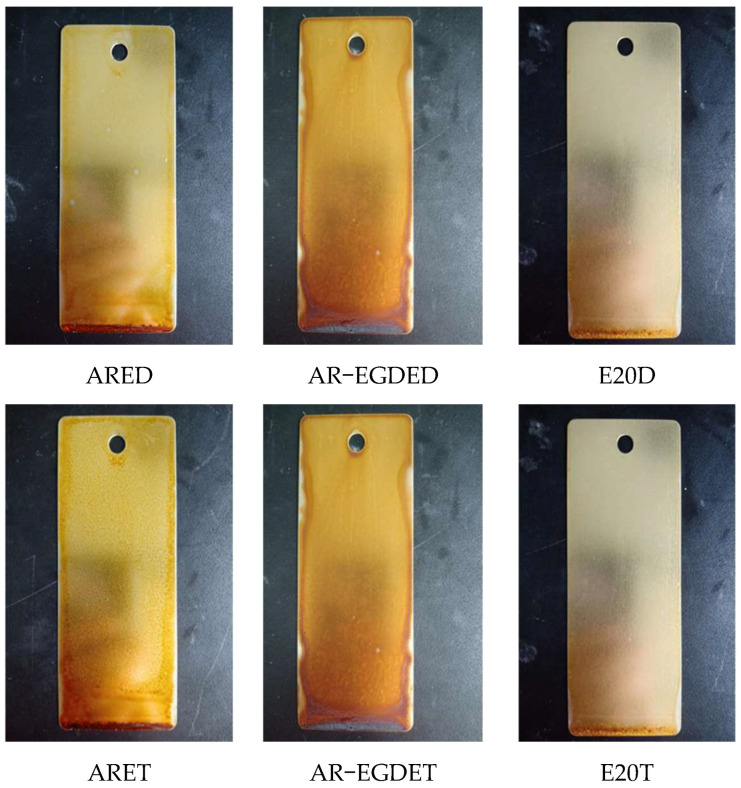
The images of the samples after heat resistance.

Figure 7 depicts photographs of each epoxy coating heated in an oven at 100 °C for three days. Based on Figure 7, the AR-EGDE coatings were the darkest brown, followed by the ARE coatings in golden yellow and the E20 in pale yellow when the same curing agent was used. The lower the heat resistance, the darker the color. Accordingly, the heat resistance decreases with increasing flexible chains and decreasing rosin-based fused rings, indicating that the fused ring structure has some heat resistance, but its heat resistance at 100 °C is inferior to that of bisphenol A epoxy resin. When the epoxy monomers are identical, there is no significant color difference between the ARED and ARET, E20D, and E20T coatings. This indicates that changes in the flexible chain of the curing agent have no effect on heat resistance. However, AR-EGDED has a slightly darker hue than AR-EGDEDT, indicating that the longer the flexible chain length, the greater the heat resistance of AR-EGDEDT.

**Figure 8:**
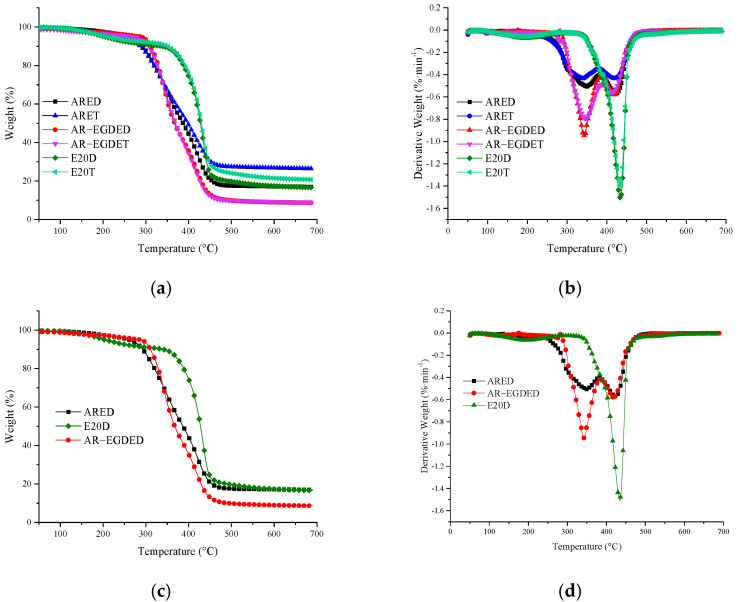
Thermal degradation curve of epoxy resins: (**a**) TG curves of epoxy resins; (**b**) D TG curves of cured resins; (**c**) TG curves of epoxy resins from DETA; (**d**) DTG curves of epoxy resins from DETA.

In addition, TGA was used to evaluate the thermal degradation of the material. Figure 8 depicts the TG and DTG as diagrams. From the TG curve, the temperature at which degradation begins (*T*_5%_) and the remaining solid residue at 700 °C were determined. From the DTG curve, the maximal weight loss rate temperatures (*T*_max_) were determined. The information is shown in Table 4. Figure 8b,d show that the *T*_max_ of rosin-based epoxy resins has two distinct peaks, and their second peak height (approximately 420 °C) is nearly identical. It suggests that the first peaks correspond to the breakdown of the flexible chain segment, while the second peaks correspond to the breakdown of the rosin-based fused ring. The first *T*_max_ of the E20 resins exhibits a lower value compared to rosin-based epoxy resins, accompanied by a notably weaker peak intensity. Consequently, the *T*_5%_ of the E20 resins is lower than that of rosin-based epoxy resin. On the other hand, the second *T*_max_ which corresponds to the degradation of the benzene ring, is higher in the E20 resins compared to rosin-based epoxy resin. This demonstrates that, in comparison to rosin-based epoxy resin, the thermal degradation of bisphenol A epoxy resin is weaker at low temperatures and stronger at high temperatures. 

### 3.5. Weather Resistance of Rosin-Based Epoxy Resin Using Monomers and Curing Agents Containing Different Amounts of Flexible Chains

**Figure 9:**
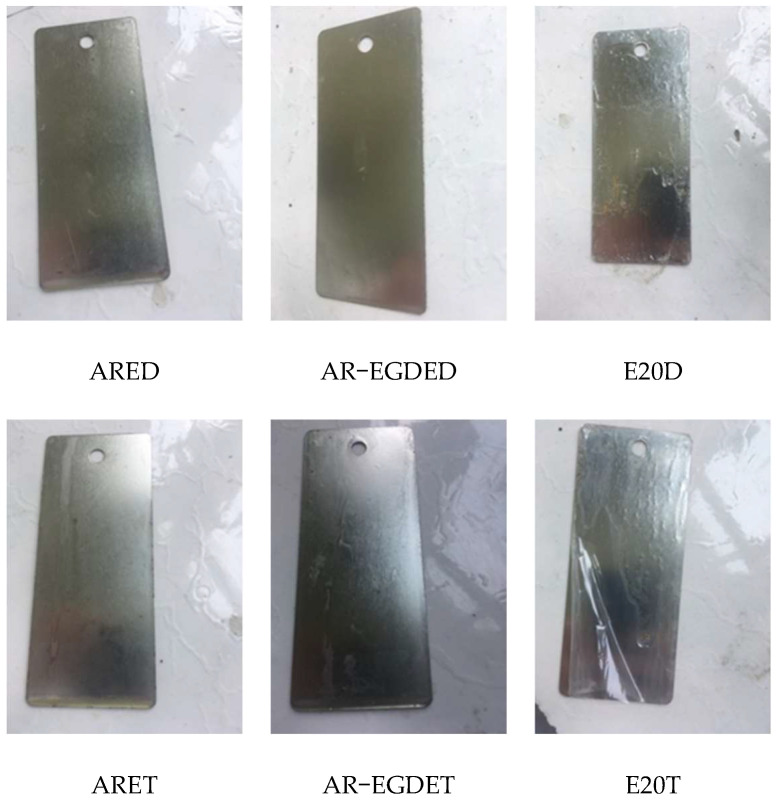
Photographs of the samples after the weatherability tests for 60 days.

Figure 9 displays photographs depicting the coatings’ conditions following the 60-day outdoor weather resistance tests. The specific outcomes of these tests are provided in Table 5. It is evident that none of the rosin-based coatings sustained any damage, whereas the E20 coatings exhibited wrinkling, flaking, and pulverization. The information suggests that the fused ring in rosin-based epoxy resin exhibits superior weather resistance properties compared to bisphenol A epoxy resin. This characteristic has the potential to compensate for the inadequate weather resistance of bisphenol A epoxy resin.

### 3.6. Effects of Flexible Chains on Properties of Rosin-Based Cured EPOXY Coating 

When the curing agent remained the same, the adhesion, flexibility, and impact resistance of the rosin-based epoxy resins improved as the flexible chain of the epoxy monomer increased from ARE to AR-EGDE. This indicates that the introduction of flexible chains decreased the brittleness of rosin-based fused rings and enhanced the toughness of rosin-based epoxy resins. When the epoxy monomer remained unchanged and the curing agent’s flexible chain grew from DETA to TETA, the performance of the resin, including its flexibility and impact resistance, remained relatively constant. This demonstrates that although the flexible chain of the curing agent was lengthened, the brittleness of the resin was not significantly reduced, so the toughness was not enhanced.

**Figure 10:**
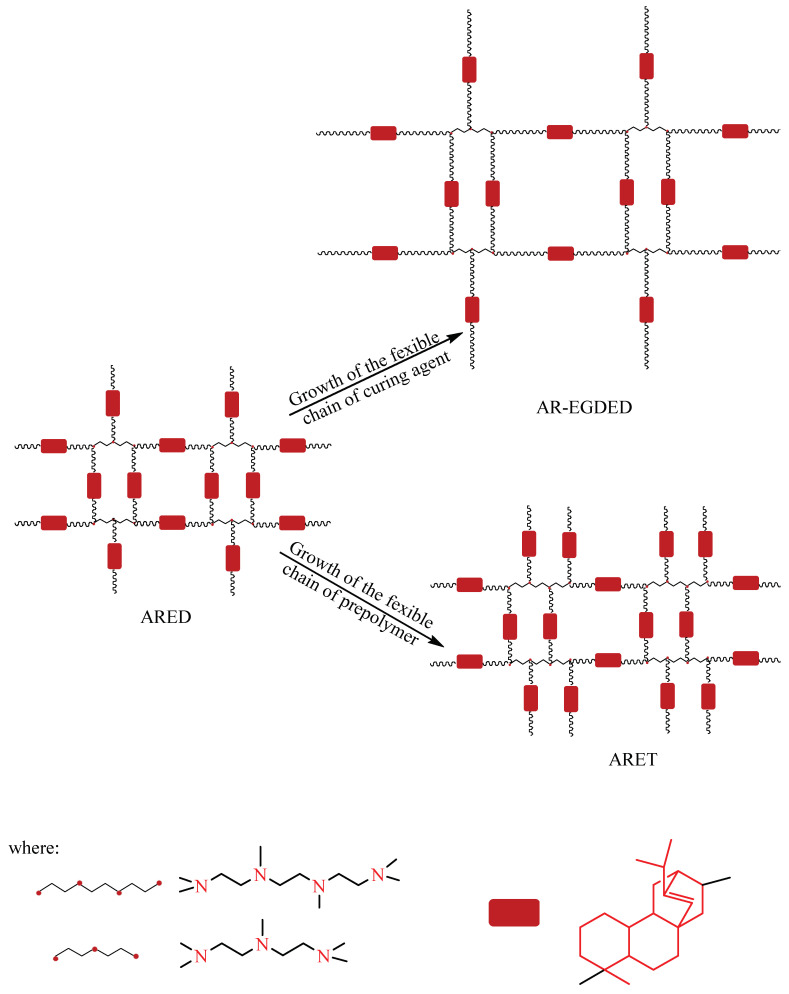
Structural diagram of acrylic rosin-based epoxy resin.

To investigate the reasons, structural fragments of three rosin-based epoxy resins were illustrated (see Figure 10). By comparing the spacing of the fused rings in ARED and AR-EGDED (rosin-based epoxy resins using the same curing agents and different monomers), it can be seen that the spacing increases with the growth of the flexible chain, whereas the spacing of ARED and ARET (rosin-based epoxy resins with the same epoxy monomer and different curing agents) changes very little with the growth of the flexible chain of the curing agent. This is because the flexible chain of the epoxy monomer did not cross-link in the resin, so the introduction of the flexible chain increased the spacing of the fused ring; the N atom in the flexible chain of the curing agent was cross-linked with the epoxy monomer containing the fused ring, which increases the number of fused rings while extending the flexible chain, resulting in a minimal change in the spacing of the fused rings. The spacing between fused rings is a significant factor in determining toughness. As the spacing widens, the molecular chain of motion increases, resulting in an improvement in toughness [35]. The spacing does not change significantly, and thus neither does the toughness.

To reduce the negative effects of the brittleness of rosin-based epoxy resin and increase its toughness, it is not necessary to simply increase the flexible chains of the epoxy monomer and curing agent; rather, the structure of the cured resin must be designed so that the rigid rosin-based fused ring is separated by an appropriate distance. 

## 4. Conclusions

The adhesion, flexibility, impact resistance, water resistance, and heat resistance of rosin-based epoxy resins containing epoxy monomers and curing agents with different flexible chains were compared. When the curing agent was left unchanged and the length of the flexible chain of the epoxy monomer was increased (from ARE to AR-EGDE), the toughness of the rosin-based epoxy resins increased, resulting in an increase in adhesion and a decrease in heat resistance. When the epoxy monomer was left unchanged and the length of the flexible chain of the curing agent was increased (from DETA to TETA), the performance of the rosin-based epoxy resin was not significantly altered. Analyzing the structure of rosin-based epoxy resin revealed that changing the epoxy monomer from ARE to AR-EGDE with a longer flexible chain increased the spacing between the rosin-based fused ring, thereby decreasing the brittleness and altering its properties. However, changing the curing agent from DETA to TETA, which has a longer flexible chain, had minimal effects on brittleness and resin performance. Therefore, when introducing a flexible chain to improve the brittleness of rosin-based resins, it is necessary to increase the spacing between the fused rings in the resins. In addition, rosin-based epoxy resin outperformed bisphenol A epoxy resin E20 in adhesion and weather resistance, allowing it to have a broader range of applications.

## Figures and Tables

**Table 1:** The formulas of epoxy resins.

Epoxy Resins	Epoxy Monomer	Curing Agent
ARED	ARE	DETA
ARET	ARE	TETA
AREP	ARE	TEPA
AR-EGDED	AR-EGDE	DETA
AR-EGDET	AR-EGDE	TETA
E20D	E20	DETA
E20T	E20	TETA

**Table 2:** Results of film performance.

Coating Sample	Pencil Hardness	Grading of Cross-Cut Tests	Flexibility	Impact Resistance
ARED	3H	3	Destroyed	Cracks and spalling
ARET	3H	3	Destroyed	Cracks and spalling
AREP	-	3	-	Cracks and spalling
AR-EGDED	2H	0	Not affected	No cracks and spalling
AR-EGDET	2H	0	Not affected	No cracks and spalling
E20D	3H	5	Not affected	No cracks and spalling, slight wrinkles and stress whitening phenomenon.
E20T	3H	5	Not affected	

**Table 3:** Water absorption rates of cured resins.

Epoxy Resins	16 h	24 h	48 h
ARED	0.24%	0.40%	0.57%
ARET	0.18%	0.39%	0.60%
AR-EGDED	5.78%	6.54%	9.35%
AR-EGDET	6.37%	7.30%	10.26%
E20D	0.26%	0.54%	1.34%
E20T	0.25%	1.14%	1.76%

**Table 4:** Thermal properties of the epoxy resins.

Cured Resins	*T*_5%_	*T*_max_	Residue at 700 °C (%)
ARED	259.93	350.27, 423.73	16.883
ARET	249.9	340.69, 419.88	26.248
AR-EGDED	287.1	343.21, 418.98	8.549
AR-EGDET	268.41	346.11, 413.81	8.612
E20D	203.94	193.69, 434.1	16.254
E20T	208.8	188.83, 433.94	20.368

**Table 5:** Results of the weatherability tests for 60 days.

Coating Sample	Observations
ARED	unchanged
ARET	unchanged
AR-EGDED	unchanged
AR-EGDET	unchanged
E20D	wrinkling, flaking, and pulverized
E20T	wrinkling, flaking, and pulverized

## Data Availability

The authors confirm that the data supporting the findings of this study are available within the article.
